# Evaluating longitudinal data accuracy in postpartum venous thromboembolism risk assessments: a retrospective observational study

**DOI:** 10.1016/j.rpth.2026.106913

**Published:** 2026-08-19

**Authors:** Marie Cahill, Fergal O’Shaughnessy, Fiona Boland, Fionnuala Ni Ainle, Jennifer Donnelly, Shane Cullinan, Brian J. Cleary

**Affiliations:** 1 School of Pharmacy and Biomolecular Sciences, Royal College of Surgeons in Ireland, Dublin 2, Ireland; 2 Pharmacy Department, The Rotunda Hospital, Dublin 1, Ireland; 3 Irish Medicines in Pregnancy Service, Rotunda Hospital, Dublin, Ireland; 4 Data Science Centre, School of Population Health, Royal College of Surgeons in Ireland, Dublin, Ireland; 5 School of Medicine, University College Dublin, Dublin 4, Ireland; 6 Department of Haematology, Mater Misericordiae University Hospital, Dublin 7, Ireland; 7 Department of Haematology, Rotunda Hospital, Dublin 1, Ireland; 8 Department of Obstetrics and Gynaecology, Royal College of Surgeons in Ireland, Dublin, Ireland

**Keywords:** postpartum period, pregnancy, risk assessment, risk factors, venous thromboembolism

## Abstract

**Introduction:**

Venous thromboembolism (VTE) risk assessment (VTERA) tools can be used to guide clinical decisions on postpartum thromboprophylaxis. However, the appropriateness of these decisions depends on the accuracy of the data input into VTERA tools.

**Objectives:**

The primary objective was to assess whether the accuracy of postpartum VTE risk assessments differed between the study years (2019-2025), across 9 predefined risk factors. The secondary objectives included evaluating the accuracy of all risk factors and thromboprophylaxis recommendations in a 2025 sample.

**Methods:**

In this retrospective observational study, the accuracy of data entered into a postpartum VTERA tool was assessed, through comparison with electronic health record data. Differences in accuracy across study years (2019-2025), for 9 structured risk factors, were evaluated using Poisson regression. For the primary analysis, all deliveries and bookings, with a matched VTERA between January 2019 and August 2025, were included. For the secondary analysis, 283 patients from 2025 were manually reviewed to assess all 23 risk factors and thromboprophylaxis recommendations.

**Results:**

Primary analysis included 47,489 deliveries and bookings, with a matched risk assessment. Body mass index was the risk factor with the lowest accuracy overall (94.3%) and remained the lowest across each year. Compared with 2019, postpartum VTE risk factor recording was significantly more accurate in 2020, 2021, 2022, 2023, 2024, and 2025. Secondary analysis showed that 6.0% of risk assessments produced inaccurate thromboprophylaxis recommendations, regarding indication and duration, and 4.9% yielded inaccurate dose recommendations.

**Conclusion:**

Future research must focus on enhancing the accuracy of VTE risk assessments. This may involve auto-population of VTERA tools using electronic health record data.

## Essentials

• Data accuracy within postpartum venous thromboembolism (VTE) risk assessment tools is unknown.
• We assessed the accuracy of postpartum VTE risk assessments between 2019 and 2025.
• Compared with 2019, VTE risk factor recording was more accurate in each subsequent year.
• In 2025, 6% of assessments gave inaccurate thromboprophylaxis indication/duration recommendations.

## 1 Introduction

Venous thromboembolism (VTE) is a leading cause of maternal morbidity and mortality worldwide [1]. The risk of VTE increases up to 60-fold in the first 1 to 2 weeks postpartum, with additional personal, pregnancy-related, and delivery-related risk factors also increasing patients’ likelihood of VTE [2,3]. Preventing maternal VTE therefore requires systematic identification of such risk factors, with VTE risk assessment (VTERA) tools assisting in this process [4,5]. VTERA tools typically require health care professionals (HCPs) to evaluate and document patient characteristics, which are known risk factors for VTE [6,7]. These risk factors can then be synthesized into a risk score that guides thromboprophylaxis.

Initially developed and validated within a general medical and surgical context, VTERA tools are now increasingly implemented in maternity care. One such maternity-specific tool is "Thrombocalc," a novel electronic VTERA tool designed to provide a standardized approach to assessing postpartum VTE risk at the point of care in a high-throughput environment [8]. Users (ie, HCPs involved in a patient’s care) enter maternal, pregnancy, and delivery risk factors into the electronic tool. Thrombocalc then generates a risk score, which determines whether thromboprophylaxis is recommended, and for what duration. The risk factors included in Thrombocalc are derived from published evidence and international guidelines [9,10].

Uptake of Thrombocalc has been high in the Rotunda Hospital, in Dublin, Ireland, since its introduction in 2014, with approximately 97% of patients undergoing assessment at the time of delivery [8]. In addition, the tool is consistently used by the same cohort of trained HCPs responsible for postpartum care. Taken together, this represents a structured and consistent approach to postpartum VTE risk assessment—creating a unique opportunity to evaluate the accuracy of risk factor identification in a large maternal population, over time, under largely optimal conditions. A 2017 study, using data from September 2014 to December 2015, described discrepancies in how Thrombocalc risk factors were recorded in the study site, with risk factor misclassification leading to inaccurate thromboprophylaxis recommendations in 2.5% of cases [8]. This phenomenon is not confined to the Rotunda Hospital. Inaccuracies in risk factor documentation within VTERA tools are an increasingly recognized concern across diverse health care systems and settings [[11], [12], [13]]. Although these discrepancies may occur for a variety of reasons—including misinterpretation, changes in status, data ambiguity, and design issues—their consequences can be profound, resulting in misclassification of VTE risk and inappropriate thromboprophylaxis [[13], [14], [15], [16]]. Indeed, a recently published Mothers and Babies: Reducing Risk through Audits and Confidential Enquiries across the UK (MBRRACE-UK) report, which examined confidential enquiries into maternal death in Ireland and the United Kingdom, revealed that HCPs often find it challenging to assess maternal VTE risk in real-life clinical practice—findings echoed by a 2022 report published by the Health Services Safety Investigations Body (HSSIB) [17,18].

It remains unclear whether the accuracy of risk factor recording, in the study site, differs between years since 2015. Accordingly, the present study compared risk assessment accuracy across the 2019-2025 study period, using specific structured risk factors as a proxy for overall accuracy. Two categories of risk factors are contained within the Thrombocalc VTERA tool: structured and unstructured risk factors (Table 1). Structured risk factors are variables that can be reliably identified in structured fields within the electronic health record (EHR), including age 35 years or older, body mass index (BMI), cesarean section, instrumental delivery, multiple pregnancy, postpartum hemorrhage (PPH), preterm birth, and stillbirth in current pregnancy. Unstructured risk factors are variables where a structured EHR data field is not available or where clinical interpretation is required.

**Table 1:** All structured and unstructured risk factors present in Thrombocalc, alongside their definitions.

No.	Risk factor	Definition	Structured?
1	Age ≥35 y	Current maternal age	Yes
2	BMI	Current BMI (kg/m^2^)	Yes
3	Cesarean section	Classified as planned or emergency cesarean section	Yes
4	Current smoker	Cigarette smoking around the time of delivery	
5	Gross varicose veins	Gross or symptomatic varicose veins that are associated with phlebitis, pain, edema, or skin changes	
6	High-risk family history	High-risk family history of VTE includes VTE occurring in a family member (or members) at a young age (typically <50 years) either unprovoked, or in the context of a minor provoking factor (eg, OCP use, pregnancy, and long distance travel)	
7	Immobility	Prolonged bed rest with no mobilization or prolonged seated immobility, for example, wheelchair user or after a long haul flight	
8	Instrumental delivery	Vaginal delivery assisted by forceps or vacuum	Yes
9	IUGR	Small for gestational age or birth weight less than the 5th centile	
10	LMWH treatment until delivery	Meets criteria for antenatal pharmacologic thromboprophylaxis or pharmacologic thromboprophylaxis prescribed in this pregnancy for the prevention of VTE	
11	Manual removal of placenta	Procedure to manually remove the placenta from the uterus after delivery	
12	Multiple pregnancy	Current pregnancy involving 2 or more fetuses (eg, twins and triplets)	Yes
13	Parity ≥3	Parity of 3 or more at booking	Yes
14	Postpartum hemorrhage >1000 mL or receipt of blood transfusion	Postpartum hemorrhage >1000 mL or receipt of a blood transfusion	Yes
15	Pre-eclampsia	Gestational hypertension: ≥140/90 mm Hg on 2 occasions ≥4 hours apart with proteinuria of at least 300 mg in a 24-hour urine collection or protein:creatinine ratio >30, arising *de novo* after the 20th week of gestation in a previously normotensive woman	
16	Preterm birth	Delivery at ≤37 weeks’ gestation in current pregnancy	Yes
17	Prolonged labor >12 h	Continuous partogram lasting >12 hours	
18	Significant medical comorbidity	The presence of a severe comorbidity for which the woman receives specialist care, for example, ulcerative colitis, systemic lupus erythematosus, Crohn’s disease, cardiac disease, and sickle cell anemia	
19	Stillbirth in current pregnancy	Intrauterine fetal death occurring at ≥24 weeks’ gestation in the current pregnancy	Yes
20	Systemic infection or COVID-19 infection	Proven systemic infection, for example, chorioamnionitis, pyelonephritis, pneumonia, and bacteremia. SIRS as defined by early warning score. Excludes less serious infections, for example, sinusitis, intrapartum fever with no proven infection	
21	Thrombophilia	Acquired or inherited (eg, factor V Leiden, protein C deficiency, protein S deficiency, antithrombin deficiency, prothrombin gene mutation, and antiphospholipid syndrome)	
22	VTE before this pregnancy	Women with previous VTE (except those with a single previous VTE related to major surgery and no other risk factors)	
23	VTE in current pregnancy	Objectively confirmed VTE (deep vein thrombosis or pulmonary embolism) occurring at any stage during the current pregnancy before delivery	

BMI, body mass index; IUGR, intrauterine growth restriction; LMWH, low-molecular-weight heparin; OCP, oral contraceptive pill; SIRS, systemic inflammatory response syndrome; VTE, venous thromboembolism.

The overall accuracy of current VTE risk assessment, including all risk factors, will be evaluated through manual review of a sample of assessments from 2025. Concordance between the resulting thromboprophylaxis recommendations and local guidelines will also be assessed within this subset. Such work will help to inform the development of targeted clinical interventions designed to optimize VTE prevention practices, while assessing the real-world impact of changes implemented at the study site since the tool was first introduced.

## 2 Objectives

The primary objective was to assess whether the accuracy of postpartum VTERAs differed across the 2019-2025 study period, using 9 predefined risk factors (as a proxy for overall accuracy over time). The secondary objectives included evaluating the accuracy of completed risk assessments (including all risk factors) in a sample of risk assessments completed in 2025 and evaluating the accuracy of thromboprophylaxis recommendations in the above subset.

## 3 Methods

### 3.1 Study design

Our study was carried out in the Rotunda Hospital, which is a tertiary referral maternity hospital in an urban setting. Thrombocalc has been in place in this institution since September 2014. An Oracle Health Millennium EHR has been in use since 2017.

In this retrospective observational study, we assessed the accuracy of data entered into Thrombocalc by comparing risk factors entered into VTERAs by HCPs with data extracted from the hospital’s EHR system. Risk factor accuracy was defined as agreement between risk factor data entered into the Thrombocalc risk assessment and the corresponding data recorded in the EHR. The accuracy of completed risk assessments (including all risk factors), as well as thromboprophylaxis recommendations, was evaluated by comparing them with risk assessments completed by, and recommendations generated by, an independent reviewer.

Thrombocalc is a bespoke postpartum HTML VTERA tool with basic Health Level Seven integration to the hospital’s patient management system. Completing the Thrombocalc risk assessment generates a risk score. As the tool applies predefined score ranges, different scores may yield the same prophylaxis recommendation if they fall within the same category. Thrombocalc also provides the appropriate weight-adjusted low-molecular-weight heparin (LMWH) dose, calculated according to maternal weight and corresponding dosing thresholds in line with clinical guidelines. This study was approved by the Rotunda Research Advisory Group (RAG-2025-004).

### 3.2 Study population

All women who booked for care, had a delivery, and had a postpartum VTERA completed between January 2019 and August 2025 were included in the primary analysis of 9 predefined risk factors.

A subsample of 283 patients from 2025 was selected for detailed manual review to assess all 23 risk factors and thereby enable evaluation of thromboprophylaxis recommendations regarding indication and duration, alongside LMWH dose recommendations. The Thrombocalc reassessment rate (ie, whereby multiple assessments are completed for the same delivery, for the same patient) was also calculated by an independent reviewer. These reassessments were quantified, as a validation step, to ensure that the most up-to-date risk assessment was used for the manual review portion of the study.

A second reviewer (F.O.S.) was assigned 30 randomly selected patient charts from the 283 manually reviewed patients (10.6%). This reviewer completed risk assessments for these patients and assigned thromboprophylaxis recommendations for comparison with the primary reviewer (M.C.). Any disagreements were resolved by discussion.

### 3.3 Data collection

Data were obtained from 2 sources: the Thrombocalc VTERA form completed by the original users and the corresponding patient EHR. For the primary analysis, structured EHR reports were used to extract structured risk factors, which were compared with corresponding entries in previously completed VTERAs. For the secondary analysis, manual review of all relevant clinical data recorded in the EHR was conducted so as to extract unstructured and structured risk factors, which were then compared with corresponding VTERAs.

### 3.4 Sample size

The primary analysis included all women who booked for care, had a delivery, and had a risk assessment between January 2019 and August 2025. Assuming a 5% inconsistency rate across structured risk factors, this sample size allows estimation of the proportion of deliveries with inconsistencies, with a margin of error of <1% at the 95% confidence level.

For the secondary analysis, assuming that a clinically relevant inconsistency (across all 23 risk factors) occurs for 10% of deliveries, a sample size of 283 allows estimation of this proportion with a margin of error of 3.5% at the 95% confidence level.

### 3.5 Statistical analysis

Initially, descriptive statistics were calculated for each risk factor. For binary variables (ie, age 35 years or more, instrumental delivery, multiple pregnancy, parity of 3 or more, PPH, preterm birth, and stillbirth), counts, percentages, and proportions of match/mismatch between Thrombocalc and the EHR were reported. For nominal variables (ie, BMI and cesarean section), frequency distributions and cross-tabulations were generated to describe agreement between Thrombocalc and the EHR.

To formally assess agreement between Thrombocalc and the EHR, McNemar’s test was used for paired binary variables testing the null hypothesis that the proportions of discordant pairs are equal (Thrombocalc = 1 and EHR = 0 vs Thrombocalc = 0 and EHR = 1). Cohen’s kappa was used to measure agreement for nominal variables beyond chance. The null hypothesis assumed no agreement beyond chance between the 2 data sources.

To evaluate differences in risk assessment accuracy between study years, modified Poisson regression with cluster-robust standard errors was fitted. The dependent variable was a binary indicator of overall assessment accuracy, defined as 1 if all 9 risk factors were accurately documented and 0 if at least 1 was inaccurate. Given that the outcome was common, this approach was used to directly estimate risk ratios for a common binary outcome, using methods described by Zou [19]. Models were adjusted for potential confounding variables (including time of delivery and number of deliveries per day). As some women contributed more than 1 pregnancy, cluster-robust standard errors were applied at the patient level. Statistical analysis was completed using R version 4.4.3.

For the manually reviewed subsample, descriptive statistics were performed (presented with Agresti-Coull confidence intervals), alongside Cohen’s kappa to assess agreement between thromboprophylaxis recommendations generated by the original clinical users and the recommendations judged appropriate based on local VTE prevention policies.

## 4 Results

During the study period, a total of 61,192 Thrombocalc risk assessments were completed, and 51,617 patients booked and delivered in the study site. Of these, 3279 were excluded because they did not possess the required data (ie, patients’ BMI was not recorded), resulting in 48,338 eligible patients who had booked and delivered. Of those 48,338 eligible patients (who booked and delivered), 47,489 were successfully matched to a corresponding Thrombocalc risk assessment, representing 92.0% of all deliveries and bookings that occurred in the study period (see the Figure).

**Figure:**
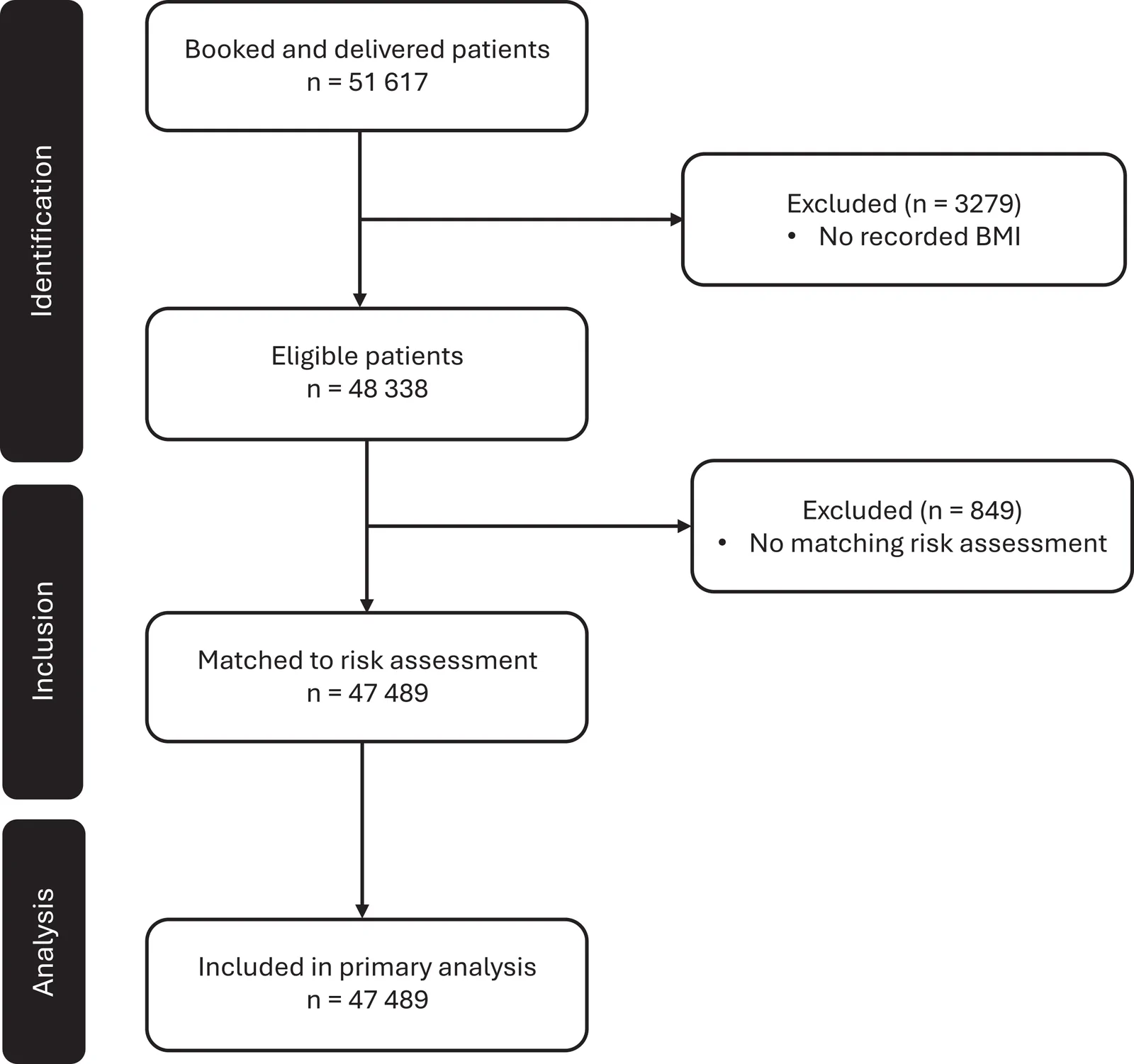
Strengthening the Reporting of Observational Studies in Epidemiology (STROBE) flow chart of the study population.

The higher number of risk assessments, relative to patients who booked and delivered, reflects the frequency with which Thrombocalc reassessments are conducted at the study site. These reassessments were handled by choosing the most recently completed Thrombocalc within 6 days of the delivery date.

### 4.1 Structured risk factor accuracy across study years

Table 2 shows the percentage of accurately recorded structured risk factors (*n* = 9), stratified by year. The structured risk factor with the highest overall accuracy was stillbirth, followed by multiple pregnancy—both of which were associated with a very high level of accuracy each year between 2019 and 2025 (ie, over 99.4%). BMI had the lowest recording accuracy overall and remained the lowest in each individual year. Patients with a BMI of 40 or more were most often accurately classified (97.5% overall). A total of 2.6% of patients with a BMI of <25 were misclassified as having a BMI of 25 to 29.9. The total number of risk assessments, containing at least 1 inaccurately recorded structured risk factor, was 7071 (14.9%; 7071 of 47,489).

**Table 2:** Accuracy counts and percentage accuracy per structured risk factor per year.

Structured risk factor	2019	2020	2021	2022	2023	2024	2025	Overall
Age ≥ 35 y, % (*n*/*N*)	97.7 (4143/4240)^a^	98.5 (7544/7659)	98.4 (7876/8001)	98.0 (7493/7643)	98.3 (7520/7648)	98.2 (7399/7533)	98.2 (4680/4765)	98.2 (46,655/47,489)
BMI, % (*n*/*N*)	94.4 (4002/4240)	95.7 (7330/7659)	94.3 (7545/8001)	94.8 (7244/7643)	92.6 (7083/7648)	93.8 (7063/7533)	94.7 (4512/4765)	94.3 (44,779/47,489)
C-section, % (*n*/*N*)	97.5 (4136/4240)	98.1 (7516/7659)	98.1 (7852/8001)	98.2 (7507/7643)	97.9 (7489/7648)	98.3 (7407/7533)	98.7 (4705/4765)	98.2 (46,612/47,489)
Instrumental delivery, % (*n*/*N*)	98.4 (4174/4240)	98.5 (7547/7659)	98.6 (7891/8001)	98.6 (7535/7643)	98.8 (7559/7648)	99.0 (7457/7533)	99.0 (4716/4765)	98.7 (46,879/47,489)
Multiple pregnancy, % (*n*/*N*)	99.5 (4220/4240)	99.7 (7634/7659)	99.8 (7982/8001)	99.7 (7623/7643)	99.8 (7630/7648)	99.7 (7508/7533)	99.6 (4748/4765)	99.7 (47,345/47,489)
Parity, ≥ 3, % (*n*/*N*)	98.4 (4021/4240)	98.0 (7503/7659)	98.4 (7869/8001)	98.2 (7507/7643)	98.5 (7531/7648)	98.7 (7432/7533)	98.7 (4703/4765)	98.1 (46,566/47,489)
PPH, % (*n*/*N*)	97.8 (4146/4240)	97.8 (7493/7659)	98.2 (7860/8001)	97.9 (7481/7643)	98.1 (7506/7648)	98.4 (7410/7533)	98.5 (4692/4765)	98.1 (46,588/47,489)
PTB, % (*n*/*N*)	97.9 (4149/4240)	98.4 (7537/7659)	98.6 (7891/8001)	98.4 (7522/7643)	98.5 (7533/7648)	98.7 (7438/7533)	98.9 (4713/4765)	98.5 (46,783/47,489)
Stillbirth, % (*n*/*N*)	99.9 (4234/4240)	99.9 (7648/7659)	99.9 (7996/8001)	99.9 (7635/7643)	99.9 (7639/7648)	99.9 (7527/7533)	99.9 (4762/4765)	99.9 (47,441/47,489)

BMI, body mass index; C-section, cesarean section; PPH, postpartum hemorrhage; PTB, preterm birth.

a The lower number of patients reported in 2019 reflects the fact that many patients who delivered that year had their booking visit in 2018 and therefore could not be included in the analysis.

There were 7 binary risk factors (of the 9 structured risk factors): age 35 years or more, instrumental delivery, multiple pregnancy, parity of 3 or more, PPH, preterm birth, and stillbirth. McNemar’s test demonstrated systematic disagreement between the true values of the 7 binary risk factors and data entered by original users (Table 3). Very large χ^2^ values were obtained for preterm birth (565.76) and instrumental delivery (326.09), indicating that the discordant pairs were heavily imbalanced and that clinicians tended to misreport in 1 direction. For example, PPH tended to be over-reported in Thrombocalc risk assessments, whereas preterm birth tended to be under-reported. When examined on a yearly basis, all 7 binary-structured risk factors showed a statistically significant disagreement between true values and what was entered each year, with the exception of stillbirth in 2019, 2023, 2024, and 2025, and parity in 2022 and 2025.

**Table 3:** Results of McNemar’s test for binary-structured risk factors, comparing true values with values entered into Thrombocalc.

Binary-structured risk factor	McNemar’s χ^2^	*P* value	Under- or over-reported in TC	Risk factor assigned in TC but not in EHR	Risk factor assigned in EHR but not in TC
Age ≥ 35 y	207.5	<.001	Under	209	625
Instrumental delivery	326.09	<.001	Under	82	528
Multiple pregnancy	100	<.001	Under	12	132
Parity ≥ 3	72.677	<.001	Over	591	332
PTB	565.76	<.001	Under	37	669
PPH	239.98	<.001	Over	683	218
Stillbirth	27	<.001	Under	6	42

EHR, electronic health record; PPH, postpartum hemorrhage; PTB, pre-term birth; TC, Thrombocalc.

Cohen’s kappa indicated high overall agreement for the 2 categorical structured variables, BMI and cesarean section, whereby cesarean section had a kappa of 0.982 (*z*-score = 214, *P* <.05) and BMI achieved a kappa of 0.951 (*z*-score = 207, *P* <.05). When further stratified by year, each category remained statistically significant, with agreement remaining consistently high overall, across all years.

The outputs of the multivariable Poisson regression model can be seen in Table 4.

**Table 4:** Outputs from the Poisson regression model, evaluating the probability that all 9 structured risk factors (RR) were accurately assigned, having adjusted for the number of deliveries, time of day, and within-woman clustering.

Year	RR	95% CI	Interpretation
2020	1.06	1.04-1.08	6.2% higher probability of all 9 risk factors being accurately assigned than in 2019
2021	1.06	1.05-1.08	6.4% higher probability than 2019
2022	1.05	1.04-1.07	5.4% higher probability than 2019
2023	1.04	1.02-1.06	3.9% higher probability than 2019
2024	1.07	1.05-1.08	6.5% higher probability than 2019
2025	1.09	1.07-1.10	8.6% higher probability than 2019

Compared with 2019, the probability that all 9 structured risk factors were accurately assigned was significantly higher in every subsequent year, even after adjusting for the number of deliveries, time of day, and within-woman clustering. The year 2023, however, demonstrated a slightly smaller improvement (3.9%) than 2020 (6.2%), 2021 (6.4%), 2022 (5.4%), 2024 (6.5%), and 2025 (8.6%).

There was no evidence that the number of deliveries on a given day influenced the probability of all 9 risk factors being accurate. Assessments performed outside normal working hours (between 20.00 and 08.00 hours), however, had a 1.8% higher probability of being accurate, compared with those performed during working hours (between 08.00 and 20.00 hours), after adjustment for year and number of deliveries.

### 4.2 Manual review subsample

#### 4.2.1 Unstructured risk factor accuracy

Thirty randomly selected patient charts from the 283 manually reviewed patients (10.6%) were assessed by a second reviewer to compare with the primary reviewer. The inter-rater reliability between the 2 manual reviewers was assessed with Cohen’s kappa. The weighted kappa was 0.92 [20].

A descriptive summary of the percentage of inaccurately recorded unstructured risk factors is presented in Table 5 (calculated using the manually reviewed subsample from 2025; *n* = 283). The unstructured risk factors with the lowest accuracy were current smoker and gross varicose veins (97.5%; 276 of 283), whereas the risk factors with the highest accuracy were immobility and VTE in current pregnancy (100%; 283 of 283).

**Table 5:** Accuracy counts and percentage accuracy per unstructured risk factor in 2025.

Unstructured risk factor	Accuracy *n* (%)
Current smoker	276 (97.5)
Gross varicose veins	276 (97.5)
High-risk family history	282 (99.6)
Immobility	283 (100)
IUGR	278 (98.2)
LMWH treatment until delivery	282 (99.6)
Manual removal of placenta	282 (99.6)
Pre-eclampsia	282 (99.6)
Prolonged labor >12 h	274 (96.8)
Significant medical comorbidity	280 (98.9)
Systemic infection or COVID-19 infection	280 (98.9)
Thrombophilia	279 (98.6)
VTE in current pregnancy	283 (100)
VTE before this pregnancy	281 (99.3)

IUGR, intrauterine growth restriction; LMWH, low-molecular-weight heparin; VTE, venous thromboembolism.

#### 4.2.2 Thromboprophylaxis recommendation accuracy

When all risk factors were considered, the number of risk assessments, with at least 1 risk factor inaccuracy, was 73 (out of 283; 25.8%). This is reflected in the VTE risk score, which was inaccurate for 63 risk assessments (out of 283; 22.3%).

A total of 6.0% (95% CI, 3.7%-9.5%) of risk assessments (17 of 283) from the 2025 sample produced inaccurate thromboprophylaxis recommendations (regarding whether thromboprophylaxis was indicated, and for what duration). Nine of these assessments were overestimations of thromboprophylaxis required, whereas 8 were underestimations. A total of 4.9% (95% CI, 2.9%-8.2%) of risk assessments (14 of 283) were found to have yielded inaccurate LMWH dose recommendations.

Twenty-five (8.8%) risk assessments were reassessments, whereby more than 1 Thrombocalc was completed for a single patient’s delivery. Twenty-two of these risk assessments were completed twice and 3 were completed 3 times.

Cohen’s kappa showed overall high agreement between appropriate thromboprophylaxis recommendations and recommendations generated by the original users (kappa = 0.92, *z*-score = 15.6, *P* <.05). The same was also found regarding LMWH dose recommendations (kappa = 0.918, *z*-score = 15.4, *P* < .05).

#### 4.2.3 Sensitivity analysis

On reviewing the 17 risk assessments that were classified as having inaccurate recommendations, 8 (47%) involved at least 1 unstructured risk factor and 9 (53%) involved only structured risk factors, where the reference standard is more objective. As a sensitivity analysis, we considered a conservative worst-case scenario in which all 8 discrepancies involving unstructured risk factors were due to misclassification in the reference-standard review rather than true tool errors. Under this assumption, the thromboprophylaxis recommendation inaccuracy rate would decrease from 6.0% to 3.2%. This represents the maximum plausible impact of uncertainty associated with unstructured risk factors and suggests that the overall findings are not solely explained by challenges in interpreting unstructured EHR data.

## 5 Discussion

Compared with 2019, postpartum VTE risk factor recording was more accurate in 2020, 2021, 2022, 2023, 2024, and 2025. We also found that 6.0% of risk assessments produced inaccurate thromboprophylaxis recommendations, regarding indication and duration, whereas 4.9% of assessments yielded inaccurate dose recommendations. To our knowledge, this is the first study to compare the accuracy of postpartum VTE risk factor recording across multiple years. Our findings regarding overall thromboprophylaxis recommendation accuracy represent an update to previous work completed at the study site, where a lower inaccuracy rate was reported.

A number of studies have evaluated the accuracy of VTE risk assessment outputs in real-world clinical practice. Pannucci et al. [21] found that 37% (848 of 2285) of patients had their VTE risk scores underestimated by clinicians, when comparing computer-calculated Caprini scores with physician-reported scores. Kim et al. [16] reported that, when comparing admitting services with a VTE consult service, 13% (13 of 100) had incorrect VTERAs completed—with incorrect thromboprophylaxis being ordered for 8 patients, as a result. Lau et al. [13] showed that 34.7% (1396 of 4021) of medically ill patients were inaccurately assessed for their VTE risk on admission to hospital—resulting in inappropriate thromboprophylaxis regimens being recommended, when comparing subject matter expert’s assigned scores with those obtained by admitting HCPs. Hoyer at al. [11] found that initial mobility assessments conducted by admitting HCPs as part of their VTERA process did not accurately reflect mobility because reduced mobility identified by researchers was associated with thrombotic events, whereas reduced mobility recorded by admitting HCPs was not. These studies reveal clear issues in how clinicians assign VTE risk factors and the potential impact on thromboprophylaxis use—findings that mirror our own.

BMI was the most frequently misassigned risk factor in our study, with users being most likely to miscategorize patients with a BMI between 25 and 29.9. Categorizing patients as having a BMI between 25 and 29.9 assigns an additional 1.5 points to their total VTE risk score, potentially altering their thromboprophylaxis recommendation if this changes their overall risk category. The presence of distinct weight fields, within the EHR, for dosing weight, measured weight, and estimated weight, may contribute to incorrect BMI categorization when completing a risk assessment. Indeed, multiple weights may be recorded for a single patient during their pregnancy—especially in patients with conditions such as gestational diabetes—which may also lead users to select the incorrect weight as the basis for patients’ BMI. In addition, BMI categories are presented within a drop-down menu in the VTERA tool, potentially creating selection errors for this risk factor. Meanwhile, stillbirth was the most often accurately identified risk factor, closely followed by multiple pregnancy. This may be due to the more evident nature of these risk factors, which typically do not require users to search through a patient’s EHR, in order to correctly assign them.

The probability that all 9 risk factors were accurately assigned was significantly higher in every subsequent year after 2019, although these differences were not consistent across all years. For example, compared with 2019, the probability of accurately recording VTE risk factors was 3.9% higher in 2023 and 6.4% higher in 2021. It is, however, notable that assessments completed outside of working hours had a higher probability of being accurate. This may indicate that users experience fewer interruptions or competing clinical activities during these time windows [22,23]. Various other factors may potentially contribute to differences in VTE risk assessment accuracy between study years; however, these were not purposefully evaluated in the present study. At the study site, quality improvement initiatives relating to the design of Thrombocalc were undertaken in 2017 and 2018 before the study period. Although we initially sought to include these years, structured EHR reports could not be generated reliably before 2019—necessitating restriction of the analysis to the 2019-2025 study period. Provider education has occurred throughout the study period typically through informal peer-to-peer training of new staff. More recently, an EHR-integrated, semi-automated prototype of the VTERA tool was developed and evaluated in 2026; however, as this prototype has not yet been implemented in routine clinical practice, it did not affect the findings of the present study.

We identified a higher overall inaccuracy rate, regarding thromboprophylaxis recommendations, when compared with previous work conducted by O’Shaughnessy et al. using 2014 to 2015 data [8]. The reasons for this are likely multifactorial, including increased medical complexity among patients, as well as increased clinical activity across the study site [24,25]. The previously mentioned HSSIB report highlighted how health care staff can struggle to conduct VTE risk assessments in the complex and busy environment of postnatal wards, with competing demands and distractions potentially leading HCPs to prioritize efficiency over the thoroughness of risk assessments [18]. In addition, in the previous study, the source data for comparison with risk assessments were paper charts, whereas this study compared risk assessments with EHR data. It is possible that the increasing complexity and volume of available data could also contribute to the decreased accuracy of risk assessments.

More broadly, our study shows that the accuracy of VTE risk assessments should not be assumed (even when an established risk assessment tool is embedded in routine clinical practice, as is the case in our study site) and may differ in routine clinical practice—while also revealing challenges intrinsic to postpartum VTE assessment. Risk assessment tools aim to promote evidence-based decision-making and standardize these decisions. However, thoroughly assessing patients’ risk of VTE in the postpartum period, specifically, often requires HCPs to consider a range of risk factors that differ in how they are identified and documented. Moreover, many VTE risk factors are dynamic and can emerge throughout the postpartum period. This is reflected within Thrombocalc but also when using the Royal College of Obstetricians and Gynaecologists 2015 risk assessment model, which incorporates a number of maternal and medical risk factors to be identified by users [9]. Such complexity can create opportunities for incomplete or inaccurate application of risk assessment tools in practice and is an important limitation of postpartum VTE risk assessment [26].

As previously mentioned, the high uptake of this VTERA tool—coupled with its consistent use by trained HCPs involved in postpartum care—reflects a promising approach to postpartum VTE risk assessment. Despite this, inaccuracies in risk factor documentation persist. Therefore, future studies aiming to link maternal VTE risk factors to real-world VTE outcomes should prioritize careful evaluation of data accuracy to promote the validity of any conclusions drawn.

### 5.1 Limitations

We conducted a manual review of a small sample of patients, within a single site, and so our findings may not be generalizable to all populations and settings. In particular, the Rotunda Hospital is a specialist tertiary-referral maternal-fetal unit, thereby caring for a higher proportion of high-risk pregnancies. As such, there may be a higher prevalence of risk factors in our study site compared with other maternity units, resulting in potentially more room for errors to occur and thus more inaccurate risk assessments.

Our use of EHR data, as a “gold standard” comparator to data entered into Thrombocalc, may also be considered a limitation because of the underlying assumption that all data entered into EHRs are correct. It is possible that clinical information, such as smoking status or the presence of gross varicose veins (that would alter VTE risk scoring), was not always perfectly captured in patients’ EHRs and so may be incorrectly omitted/assigned. The potential impact of using EHR data as a comparator was explored in more detail in the above sensitivity analysis.

### 5.2 Future research

Future work should focus on systems-based approaches to ensuring validity of data entered into VTERA tools and reducing cognitive load for users. Indeed, the observed variation in accuracy across individual risk factors indicates that errors are likely influenced not only by user performance but also by the design of the tool.

Although objective risk factors that are routinely documented may be more readily identified by users, variables lacking explicit definitions within the tool (such as gross varicose veins and current smoker) may be more vulnerable to inter-user variation. The VTERA tool also requires clinicians to select only those risk factors deemed applicable, while allowing the assessment to be completed without indicating that every risk factor has been considered. Consequently, omitted risk factors may represent either a true absence of the risk factor or a mistake during assessment. This may partially explain why more binary structured risk factors were “under-reported” by Thrombocalc users than “over-reported”. Other design features, such as the aforementioned drop-down menu for BMI category, may create selection errors for this more objective risk factor.

As previously mentioned, a semi-automated version of Thrombocalc has been developed using the Substitutable Medical Applications, Reusable Technologies (SMART) on Fast Healthcare Interoperability Resources (FHIR) framework. This prototype addresses several of the above design features by enabling auto-population of 11 risk factors (including BMI), such that users are no longer required to manually enter these risk factors into the tool. Previous research on this tool showed promising results, including improved accuracy and efficiency when completing VTE risk assessments for simulated patients and high overall usability [27]. VTE risk is dynamic and evolving [9]. For example, experiencing a PPH or developing sepsis may dramatically alter a patient’s level of VTE risk. As such, the continued development of this semi-automated tool may contribute to the use of EHR data for dynamic patient reassessment as risk factors change in the peripartum period.

Future research should examine the interplay between user demographics, such as professional role, and risk factor accuracy. Examining the specific user factors associated with inaccuracies, and how to address them, may also prove worthwhile. Future qualitative research can assist in this endeavur by examining HCP perspectives on Thrombocalc and overall EHR workflow to support accurate data entry into this tool.

The goal of VTE risk assessment and thromboprophylaxis is to decrease the incidence of postpartum VTE, a potentially fatal complication of pregnancy. However, because of the low incidence of VTE among postpartum patients, clinical validation of Thrombocalc necessitates a significant study population with sufficient outcomes. To address this, further research is being undertaken by our research group to ascertain maternal VTE outcomes and validate this VTERA tool.

Our findings support growing recognition that enhancements in postpartum VTE prevention may require reconsideration of the design of VTERA tools themselves. Although comprehensive risk assessment tools seek to capture the multifactorial nature of VTE risk, rising complexity may limit reproducibility in routine clinical practice. Recent reports from HSSIB and MBRRACE-UK have similarly recognized that HCPs find existing VTERA systems challenging to apply consistently and have called for the development of a more straight-forward and reproducible clinically-validated tool [17,18].

## 6 Conclusion

Our study reveals that the accuracy of postpartum VTERAs should not be assumed and may vary in routine clinical practice. Although VTERA tool uptake is high in our study site, future research must focus on enhancing the accuracy of risk assessments and preventing discrepancies for specific data points, such as BMI. This may take the form of auto-population using EHR data or qualitative research to examine user perspectives regarding risk assessment workflow; thereby addressing the user- and systems-based reasons for these inaccuracies.
